# The role of ventricular remodeling in the early decompensation of cardiorenal syndrome: Insight from studies with Ren-2 transgenic hypertensive rats subjected to volume overload induced using aorto-caval fistula

**DOI:** 10.1038/s41440-025-02440-4

**Published:** 2025-11-10

**Authors:** Petr Kala, Matúš Miklovič, Zuzana Honetschlägerová, Zdenka Vaňourková, Petr Kujal, Janusz Sadowski, Miloš Táborský, Barbara Szeiffová Bačová, Matúš Sýkora, Michal Šnorek, Vojtěch Melenovský, Luděk Červenka

**Affiliations:** 1 Department of Cardiology, Motol University Hospital and Second Faculty of Medicine, Charles University, Prague, Czech Republic; 2 Center for Experimental Medicine, Institute for Clinical and Experimental Medicine, Prague, Czech Republic; 3 Department of Pathology, Third Faculty of Medicine, Charles University, Prague, Czech Republic; 4 Center of Experimental Medicine, Institute for Heart Research, Slovak Academy of Sciences, Bratislava, Slovakia; 5 Department of Cardiology, České Budějovice Hospital, České Budějovice, Czech Republic; 6 Department of Cardiology, Institute for Clinical and Experimental Medicine, Prague, Czech Republic

**Keywords:** Cardiorenal syndrome, Ren-2 transgenic hypertensive rats, Volume overload, Cardiac remodeling, Cardiac functions

## Abstract

The aim of the present study was to evaluate the role of the left ventricle (LV) remodeling in the process of the transition from the compensation to the decompensation phase of cardiorenal syndrome. Ren-2 transgenic rats (TGR) with aorto-caval fistula (ACF) were used as the model of cardiorenal syndrome. Two weeks after ACF creation or sham operation, heart morphological parameters, cardiac structure and function were assessed by echocardiography and invasive pressure-volume analysis. This time point was chosen because two weeks after ACF the TGR still exhibit 100% survival rate and are in the transition phase from the compensation to the decompensation of cardiorenal syndrome. Our results at this stage show: (i) ACF TGR have already fully developed eccentric LV hypertrophy as compared with sham-operated TGR which exhibited signs of LV concentric hypertrophy; (ii) the increase in whole heart weight in ACF TGR was dominantly mediated by right ventricle (RV) hypertrophy, whereas the increase in the LV mass was minimal; (iii) ACF TGR displayed, besides bilateral ventricular dilatation, significant impairment of LV systolic functions whereas RV systolic functions were not impaired as compared with sham-operated TGR. Based on our present results, we propose that the inability of the LV to develop an appropriate hypertrophic response leads to maladaptive ventricular remodeling, which is likely a crucial factor in the process of the transition from the compensation to the decompensation phase of cardiorenal syndrome.

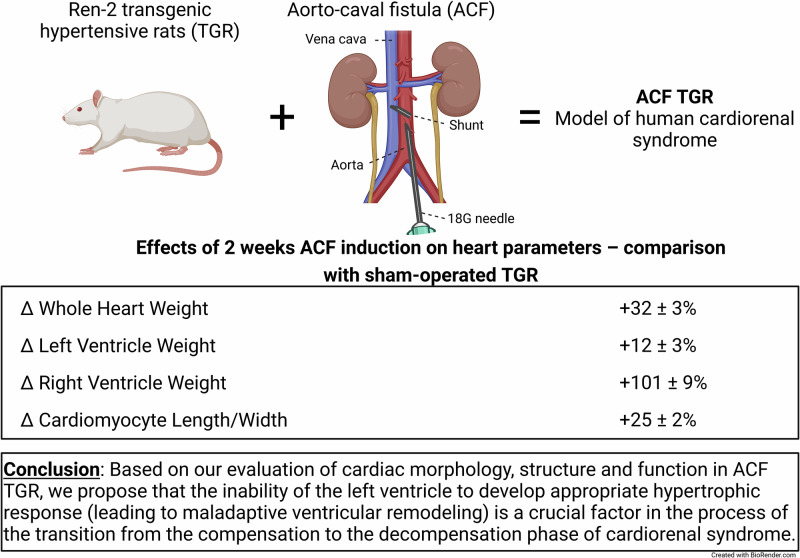

## Introduction

The profound bidirectional relationship between the kidney and the heart in the physiological regulation of cardio-renal function is now well-recognized [1–3]. The importance of this relationship in pathophysiological situations was described 190 years ago by Robert Bright [4], who was the first to describe important cardiac structural changes in patients with advanced proteinuric kidney disease. Later, 70 years ago, Barger et al. [5] demonstrated for the first time the critical role of the kidney in the pathophysiology of heart failure (HF). At present, it is recognized that if HF is accompanied by impairment of renal function, the prognosis of patients is gloomy, even worse than in most types of common cancers [6–8]. The combination of HF with renal functional impairment is called “cardiorenal syndrome”; the term has entered medical terminology over the last 20 years and has been largely accepted - obviously because of the striking connection of the syndrome with the deterioration of morbidity and mortality of patients [9–12]. Cardiorenal syndrome was initially identified by Ronco et al. [13] as “disorders of the heart and kidneys whereby acute or chronic dysfunction in one organ may induce acute or chronic dysfunction of the other”. The syndrome was divided into 5 subcategories according to the organ of origin and depending on the nature of the initial insult: acute or chronic. This description was accepted for almost two decades [10–12, 14–18]. Still, recently, the term has been conceptually modified to “chronic cardiovascular-kidney disorder” [19], which should better capture the disruption caused by disease states to organ function. Moreover, the modification was intended to achieve simplification and conceptual clarity. Regardless of the terminology used: “cardiorenal syndrome” or “chronic cardiovascular-kidney disorder”, awareness of the syndrome’s existence helped to propose adequate clinical trials and to introduce a new multidisciplinary approach in the treatment of the affected patients (”nephrocardiology”) [20–24]. Unfortunately, only scanty knowledge is available about the pathophysiological mechanisms underlying the development and particularly the progression of the syndrome. Undoubtedly, such knowledge is a prerequisite for introducing new therapeutic measures. These are urgently needed because currently available pharmacological strategies appear inadequate for effective management of the syndrome/disorder [11, 14–18, 25, 26]. Importantly, clinical investigations have demonstrated that patients with cardiorenal syndrome are known to display excessive renal sodium avidity, indicating that, at present, the research and treatment have been focused on the renal aspects of the cardiorenal syndrome [11, 15–18, 27].

We employed here the rat model of volume overload induced by the creation of the aorto-caval fistula (ACF) in Ren-2 transgenic rat (TGR), a strain that combines endogenous activation of the renin-angiotensin system (RAS) and hypertension [28, 29] - the two factors critical for the development and progression of cardiorenal syndrome [9, 10, 14, 30–32]. Earlier, we demonstrated that ACF TGR represents a reasonable model of human cardiorenal syndrome [33–38]. However, our research, in parallel with the relevant clinical studies, focused on the “kidney side,” whereas the “heart side” was to some extent disregarded. This was so even though some data regarding cardiac ventricular remodeling have suggested that in this model of the cardiorenal syndrome, the inability of the left ventricle (LV) to develop hypertrophy comparable with that of the right ventricle (RV) might be one of the critical factors responsible for the development of decompensated phase of the disorder [36–38]. Admittedly, these data were obtained either in the very early phase of cardiorenal syndrome with enduring full compensation (i.e., one week after ACF creation) or in the phase of overt decompensation (i.e. three weeks after ACF creation). Therefore, it remains unknown if the inability of LV hypertrophy to increase further is already demonstrable during the transition phase from compensation to the decompensation phase of the cardiorenal syndrome. In this context, it is important to emphasize that in the present study we use the term of cardiorenal syndrome even though renal function was not evaluated, for methodical reasons. It is currently impossible to perform simultaneous evaluation of cardiac function and renal function in the same animals; a separate series of experiments would be required. We did not perform such studies to adhere to the Russell and Burch 3 R principle [39], which is now widely recommended in animal research [40] and highly recommended by the Animal Care and Use Committee of our institute (IKEM). Thus we made use here of our data obtained in our previous studies that were exclusively focused on the “kidney side” of the cardiorenal syndrome [33, 35–37].

Accordingly, the aim of the present study was to assess heart morphological parameters and cardiac structure and function using echocardiography and invasive pressure-volume analysis of the LV, and to examine gene expression of selected genes previously implicated in the development of LV failure [41, 42]. The studies were performed two weeks after ACF creation, the time point chosen because at that stage ACF TGR were in the transition phase of the cardiorenal syndrome and still displayed a 100% survival rate. We aimed to clarify if possible changes in cardiac structure and/or function accompanied by gene expression alterations are a triggering cause of the onset of cardiorenal syndrome decompensation or are a consequence of already developed decompensation.

## Methods

### Ethical approval and animals

The study was performed in accordance with the guidelines and practices established by the Animal Care and Use Committee of the Institute for Clinical and Experimental Medicine (IKEM), Prague, which accords with the European Convention on Animal Protection and Guidelines on Research Animal Use and were approved by this committee and subsequently by the Ministry of Health of the Czech Republic (the decision number for this project is 18680/2020-4/OVZ). All animals used in the study were bred in IKEM, which is accredited by the Czech Association for Accreditation of Laboratory Animals. Experiments were performed in heterozygous TGR generated by breeding male homozygous TGR with female homozygous transgene-negative normotensive Hannover-Sprague Dawley (HanSD) rats. Homozygous breeder pairs of TGR and HanSD rats were also originally obtained from Max Delbrück Center, Berlin, Germany. The study complied with the ARRIVE (Animals in Research: Reporting In vivo experiments) guidelines [43].

### HF model and exclusion criteria

Eight-weeks-old male TGR were anesthetized with intraperitoneal ketamine/midazolam mixture (Calypsol, Gedeon Richter, Hungary, 160 mg/kg and Dormicum, Roche, France, 160 mg/kg). The HF variant dependent on volume overload was induced by creating ACF using the needle technique. This procedure is routinely performed in our laboratory, and the details of the procedure have been reported previously [33–38, 41, 42]. Sham-operated rats underwent an identical procedure but without creating ACF. Animals were excluded from the study if a technical error occurred during the ACF creation procedure or pulsatile flow in the inferior vena cava could not be confirmed, suggesting a flawed ACF function. Two weeks after creation of ACF, TGR were considered to be in the transition phase from the compensation to the decompensation of HF (cardiorenal syndrome) and were employed for the studies described below.

#### Detailed experimental design

**Fig. 1:**
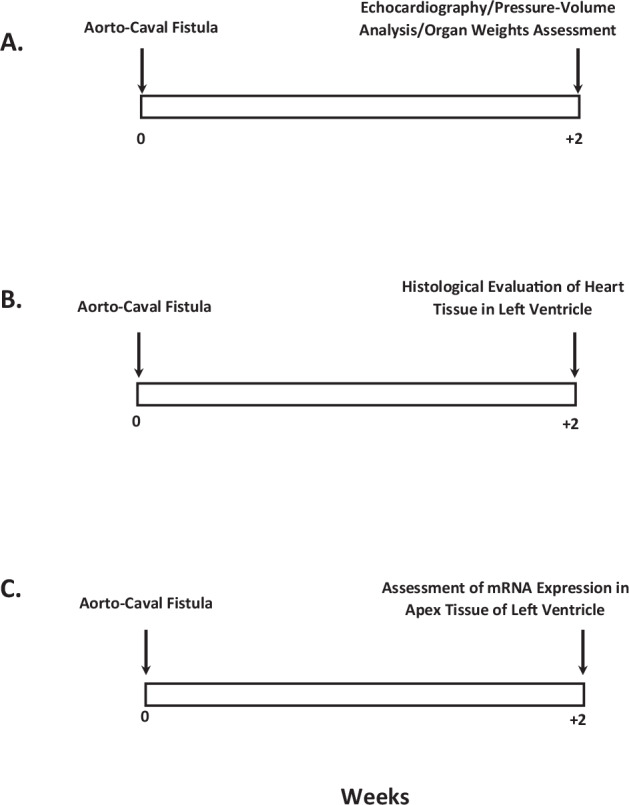
The experimental design of the whole study, delineating individual experimental series and specific functional evaluation. Series 1 focused on quantification of cardiac function, hemodynamic and organ weight changes **A**, series 2 focused on histological changes of  left ventricular tissue **B**, series 3 focused on gene expression changes in apex tissue of left ventricle **C**.

Experimental protocols for all series are outlined in Fig. 1.

### Series 1: Assessment of organ weights and cardiac structure and function by echocardiography and pressure-volume (PV) analysis in the phase of the transition from the compensation to the decompensation of cardiorenal syndrome

On the day of the experiment, rats were anesthetized with long-term anesthesia (intraperitoneal thiopental sodium, 50 mg/kg, VAUB Pharma a.s., Roztoky, Czech Republic), and echocardiography was performed as in previous studies from our laboratories [33, 41, 42, 44]. Briefly, the ventral thorax of animals was shaved, and for standard measurements of cardiac parameters, B-MODE and M-Mode images were recorded in parasternal long axis and parasternal short axis view at the papillary muscle level. Morphological parameters of the LV and right ventricle (RV), including dimension of LV and RV inner diameter, LV anterior and posterior walls, were measured in M-mode from long and short axis sections as previously described, and LV and RV cardiac functions were calculated by standard formulas [33, 41, 42, 44, 45]. All ultrasound studies were done by Vivid System (probe 10S, 5–11.5 MHz, Vivid 7 Dimension, GE Healthcare, Chicago, USA) and analyzed in EchoPAC software (version 110.1.2 GE Healthcare, Chicago, USA). The mean of three optimally obtained measurements in each rat was used for each parameter. Subsequently, LV functions were invasively assessed by employing PV analysis using techniques and protocols developed and validated for mice and rats by Pacher et al. [46]. This method was introduced almost 15 years ago in our laboratory and is routinely used in our studies evaluating cardiac functions in rats. Detailed description can be found in our numerous previous studies [33, 38, 41, 42, 44, 47]. Briefly, rats were intubated with a plastic cannula to ensure chest relaxation during the whole operation. The left jugular vein was cannulated to secure central venous access for solutions administration as required. A balloon catheter (LeMaitre Single Lumen Embolectomy Catheter, 2 F, Burlington, MA, USA) was inserted under ultrasonic control via the right jugular vein to the vena cava inferior, below the diaphragm to maintain the best position for preload reduction. Just before the PV measurement of the LV, the conductance and pressure signals of the Millar pressure-volume catheter (Millar, 2 F, Houston, TX, USA) were calibrated using MPVS software (V2.2, Millar, Houston, TX, USA) according to the manufacturer’s instructions. Functions of the LV were invasively assessed by a PV catheter introduced into the LV via the right carotid artery as described in previous studies [38, 41, 42, 44, 47]. For basal measurements, intravenous pancuronium (1 mg/kg, Inresa Arzneimittel, Freiburg, Germany) was administered through the cannulated left jugular vein and rinsed with a bolus of saline to reduce noisiness in the signal caused by breathing. To effectively determine cardiac functions, the preload reductions were performed by slowly inflating the balloon catheter placed in the vena cava inferior with aqua pour injection. The volume signal was calibrated by end-diastolic and end-systolic volume, which were obtained shortly before invasive recordings. Data from PV loops were captured and analyzed using LabChart Pro software (ADInstruments, Bella Vista, NSW, Australia). At the end of the experiment, whole heart weight (HW) and then left ventricle weight (LVW) (including septum), right ventricle weight (RVW), and lung weight (“wet lung weight”) were assessed and normalized to tibia length as described in our previous studies [34, 35, 37, 38, 48–50]. The parameters for evaluating cardiac function were calculated by employing formulas using the data obtained by echocardiography and PV analysis as originally described by Pacher et al. [46], now recognized as the “golden standard approach” in assessing cardiac systolic and diastolic function in small animals and also recently advocated by the American Physiological Society in the “guidelines paper” [51]. Therefore, this approach is standardly employed in our laboratories as documented by our previous studies [38, 47]. Here we remind some terms and formulas that are not commonly perceived by general medical community apart from professional cardiologists. Thus, maximum rates of pressure rise (+dP/dt)_max_ represent the maximum rate of pressure change in the cardiac ventricle and is traditionally used as an index of ventricular contractility. Its limitation is that it is load-dependent and thought now to be inferior to parameters defined by the PV plane, such as preload recruitable stroke work (PRSW) and end-systolic pressure-volume relationship (ESPVR). PRSW is expressed as a linear relationship between stroke work (SW) and end-diastolic volume (EDV) and is considered relatively load-insensitive so it is not significantly influenced by changes in preload and or afterload. In this context, SW is defined as the work performed by the ventricle to eject stroke volume into the aorta and is the product of stroke volume (SV) and mean arterial pressure, depicted as area enclosed by the PV loop. Mathematically, it is calculated as the integral (∫) of pressure with respect to volume, specifically the difference between ∫ of systolic blood pressure with respect to volume and the ∫ of diastolic blood pressure over volume. ESPVR describes the maximal pressure that can be developed by the LV at any given LV volume. The slope of ESPVR, known as end-systolic elastance (Ees) is an index of myocardial contractility and is, like PRSW, considered to be insensitive to changes in preload, afterload and also heart rate. The formula for the ESPVR is P = Ees * (V – V_0_), where P is the end-systolic pressure (ESP), V is the end-systolic volume and V_0_ is the volume axis intercept. Likewise, the minimum rate of pressure rate (-dP/dt)_min_ represents the minimum value of the first derivative of pressure over time in the cardiac ventricle and is employed as a valuable tool for evaluation of isovolumic relaxation. Yet again, the limitation is that it may not be a valid measure of LV relaxation rate during acute alterations in contractility and/or afterload; therefore, this parameter is currently supplemented by evaluation of isovolumic relaxation constant [Tau – (τ)]. Tau represents the exponential decay of the ventricular pressure during isovolumic relaxation and the advantage is that it is believed to be a load-independent measure of isovolumic relaxation. The Weiss method (by which τ was computed) formula is: P(t) = P0e^-(t - t₀)/τ^, where P(t) is the LV pressure at time t, P0 is the pressure at the beginning of relaxation, and τ is the time constant of isovolumic relaxation [52]. End-diastolic pressure-volume relationship (EDPVR) describes relationship in the LV between the EDV and end-diastolic pressure (EDP) at the same point, and reflects passive filling properties of the myocardium. The slope of the EDPVR at any point along this curve is the reciprocal of LV compliance. Arterial elastance (Ea) is a measure of arterial load on the LV and the formula for its calculation is: ESP/SV. LV wall stress is a measure of the force exerted by the blood on the inner wall of the LV: it is a key indicator of the mechanical load of the myocardium and can be calculated using the law of Laplace; the formula used is: wall stress = P x r/2w, where P is ESP (in our setup LV peak pressure), r is the internal radius of the LV and w is the thickness of the LV wall. In this context another important parameter that is derived from echocardiography is relative wall thickness which is a measure of LV geometry and is calculated by the formula: relative wall thickness = 2 * LV posterior wall thickness/LV diastolic diameter. Ventriculo-Arterial Coupling (VAC) is a measure of efficient interaction between ventricular contractility and the resistance it faces from arteries. The formula is: VAC = E_a_/E_es_. VAC values ˃1.0 are considered as pathophysiological, indicating hemodynamic derangements and reduced cardiac efficiency. Potential Energy (PE) refers to the energy stored within the ventricular muscle at the end of systole, which is not immediately converted into mechanical work but rather dissipated as heat during isovolumic relaxation; it is depicted as the area enclosed by the PV loop. Mathematically, it is calculated as ∫ area bound by both the ESPVR and EDPVR and the isovolumic relaxation limb of the same PV plot. The total pressure-volume area (PVA) represents the total mechanical energy produced by ventricle at each cardiac cycle and the formula is: PVA = PE + SW. It is known that PVA correlates linearly with myocardial oxygen consumption, which includes basal metabolism, intracellular calcium cycling and cross-bridge cycling, and is used for evaluating the efficiency of cardiac function. In this context, the efficiency of cardiac ventricle can expressed as the ratio of the useful ventricular SW and the oxygen consumption as estimated by PVA. The formula is: Ventricular efficiency = SW/PVA. The following experimental groups were examined:

1. Sham-operated HanSD rats (no ACF) (*n* = 10)
2. Sham-operated TGR (*n* = 10)
3. ACF TGR in the transition from the compensation phase to the decompensation phase (i.e. two weeks after the creation of ACF) (*n* = 13)

### Series 2: Histological evaluation of heart tissue in the phase of the transition from the compensation to the decompensation of cardiorenal syndrome

In separate appropriately matched (regarding the protocol as well as numbers) three experimental groups, i.e., sham-operated HanSD rats, sham-operated TGR (no ACF in both), and ACF TGR, the hearts were subjected to histological examination as described previously [36, 37, 45, 53]. The reason for the need for separate experimental groups for histological evaluation lies in the use of cardioplegia solution with subsequent immediate fixation in paraformaldehyde solution; this procedure precludes precise determination of HW, LVW, and RVW.

Briefly, two weeks after either sham operation or ACF creation, the rats were anesthetized with a combination of midazolam 50 mg.kg^-1^ (Dormicum, Roche Ltd., Prague, Czech Republic) and ketamine 50 mg.kg^-1^ (Calypsol, Gedeon Richter Ltd., Budapest, Hungry) i.p. The beating (pulsating) organ, i.e., the native heart, was perfused in situ with 20 ml of Thomas cardioplegia solution and subsequently fixed in 4% paraformaldehyde in phosphate-buffered saline and embedded into Tissue-Tek. The blocks were cut using a cryo-microtome, and cardiomyocyte width was measured in the subendocardium, mid-myocardium, and subepicardium of the LV. Cardiomyocyte length was measured only in the midmyocardium; 30 cardiomyocytes were assessed in each layer. Only the cells in which the nucleus was visible were measured to avoid underestimation. Since there were no significant differences in the cardiomyocyte width between the layers, the data from the subendocardium, midmyocardium, and subepicardium were pooled, as was also practiced by other investigators [36, 37, 54]. LV fibrosis were analyzed in sections stained with Picrosirius red (Direct Red 80, Sigma Aldrich, MO, USA) as described in detail previously [55]. The interstitial collagen was analyzed in polarized light using 10 images of the LV and RV scanned from the midmyocardium, without perivascular areas (magnification 200x, microscope Nikon eclipse Ni-E, camera Nikon DS-L3, Tokyo, Japan). The percent area of myocardial fibrosis was calculated semiquantitatively using imaging software NIS-Elements Ar (LIM, Prague, Czech Republic). In this study we employed circularly polarized light for analysis which unlike the linearly polarized light used previously allows discrimination between mature and newly synthesized collagen fibers [56].

### Series 3: Assessment of LV mRNA expression in the phase of the transition from the compensation to the decompensation of cardiorenal syndrome

In separate, appropriately matched three experimental groups (*n* = 9 in each), i.e., sham-operated HanSD rats, sham-operated TGR, and ACF TGR, the LV mRNA expression of selected genes (known as markers of myocardial stress, metabolism, inflammation, and fibrosis) was performed [41, 42]. The reason for the need for separate experimental groups for such analyses is the approach used to obtain tissue samples. Animals were killed by decapitation, and LV tissue samples that were taken from the apex of the LV were immediately harvested into liquid nitrogen and stored at −80 ^0^C until analysis. The tissue mRNA expression was determined using the standard technique described in previous studies, including ours [57–60]. The measurement of multiple mRNA expression was performed by the manufacturer’s instructions (384-well microfluidics TaqMan array cards; custom setting of selected genes; Applied Biosystems, Foster City, CA, USA). The investigated genes are listed in Supplemental Table 1, which shows the data, including abbreviations and the appropriate ID assay identification number given by the manufacturer. The relative gene expression was used to analyze changes in mRNA expression. This expression was calculated using the 2-∆∆Ct method, the most frequently used method for such experiments [57, 58]. The method directly uses the Ct (threshold cycle) information generated from a qPCR system. Ct is the cycle number where the fluorescence generated by the PCR product is distinguishable from the background noise. To calculate relative gene expression in target and reference samples, a housekeeping gene was used as the normalizer. 18S rRNA was used as the normalizer because its expression level remains relatively stable in response to any treatment [57, 61], and it meets the “Minimum Information for Publication of qPCR Experiments” guidelines as introduced by Bustin et al. [62].

ΔCt of each sample was first calculated:

$$\varDelta {Ct}={Ct}\left({gene\; of\; interest}\right)-{Ct}\left({housekeeping\; gene}\right)$$

The expression of mRNA of selected genes was related to a control group, *i.e*., HanSD rats treated with vehicle. The final results were expressed as the *n*-fold difference in gene expression of mRNA of target genes between the appropriate experimental group and control group calculated as follows:

$${n-{fold\; expression}=2}^{-\left(\varDelta {Ct\; of\; experimental\; group}-\varDelta {Ct\; of\; control\; group}\right)}$$

Subsequently, the log transformation of the data was performed to make it more symmetrical, as recommended and generally accepted for evaluation of relative gene expression results [57, 62, 63]. Thus, the values in the graphs represent log2 *n*-fold gene expression.

### Statistical analysis

Statistical analyses were performed using Graph-Pad Prism software (Graph Pad Software, San Diego, California, USA). Statistical comparison was made by one-way ANOVA followed by Tukey’s multi comparison test when appropriate, and all data were also analyzed by the D´Agostino-Pearson normality test, which is currently recommended as the best approach to quantify how far the distribution is from Gaussian distribution in terms of symmetry and shape, particularly for data obtained by qRT-PCR [63]. Pearson´s correlation coefficient was used to assess the correlation between continuous variables. The difference of linear regression slope from the line of equality (x = y) or between two datasets was tested. Values are expressed as mean ± SEM. Values of *p* below 0.05 were considered statistically significant.

## Results

### Organ weights and cardiac structure and function assessed by echocardiography and PV analysis at the transition phase of cardiorenal syndrome

#### Organ weights

**Fig. 2:**
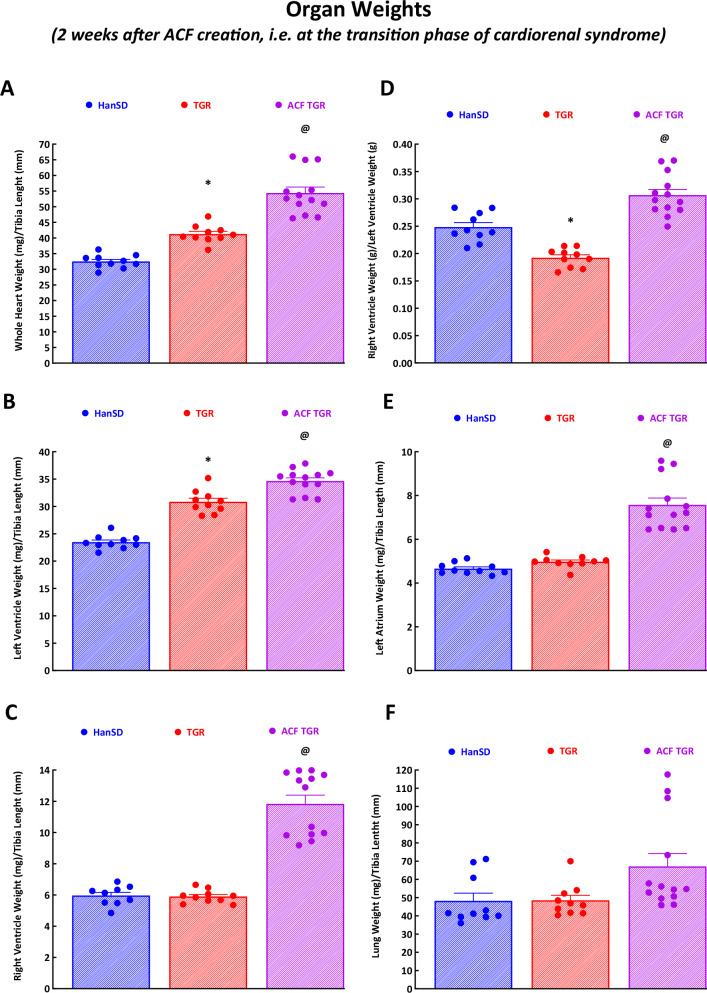
Organ weight parameters. Whole heart weight **A**, left ventricle weight **B**, right ventricle weight **C**, the ratio of right ventricle weight to left ventricle weight **D**, left atrium weight **E**, and lung weight **F** in sham-operated normotensive transgene-negative Hannover Sprague-Dawley rats (HanSD), hypertensive sham-operated Ren-2 transgenic (TGR) rats, and in the TGR two weeks after creation of the aorto-caval fistula (ACF). ^*^ *P* < 0.05 compared with sham-operated HanSD rats. ^**@**^ *P* < 0.05 compared with all other groups. The values are means ± SEM

As shown in Fig. 2A–D, sham-operated TGR exhibited discernible LV hypertrophy as seen from the whole HW and LVW (with septum) values compared to sham-operated HanSD rats (Fig. 2A, B). There was no sign of RV hypertrophy as documented by the RVW and particularly by the decreases in RVW to LVW ratio (Fig. 2D). ACF TGR exhibited a further marked bilateral cardiac hypertrophy in this phase, as seen from the whole HW, LVW, and RVW – as compared to sham-operated TGR (Fig. 2A–C). Remarkably, the increases in RV hypertrophy were in all ACF TGR discernibly higher than that of the LV - as documented by increases in RVW to LVW ratio (Fig. 2D).

As shown in Fig. 2E there were no significant differences in the left atrium weight between sham-operated HanSD rats and sham-operated TGR, however, ACF TGR displayed significantly higher left atrium weight (Fig. 2E).

Sham-operated TGR (as compared with sham-operated HanSD rats) did not show any indication of lung congestion, as documented by wet lung weight (Fig. 2F). ACF TGR did not show significantly higher lung weight than sham-operated TGR, indicating that in this phase no important lung congestion was present in the former (Fig. 2F). However, it is important to emphasize that three out of thirteen ACF TGR showed markedly higher lung weight (almost twofold higher than the group average), indicating the development of important lung congestion (Fig. 2F). In addition, those three ACF TGR showed no increase in LVW compared to sham-operated TGR (the LVW was almost identical to the average in the group of sham-operated TGR – Fig. 2B), but exhibited the greatest increases in left atrium weight as compared with sham-operated TGR (Fig. 2E).

The RV hypertrophy in those rats was more pronounced than that seen from the increases in the ratio of RVW to LVW (the said three rats exhibited the highest RVW to LVW ratio in the ACF TGR group – Fig. 2D). There were no significant differences in kidney weight between experimental groups and therefore data are not shown.

Gross morphological changes in the heart are presented in Supplemental Fig. 1, which shows representative whole heart images in sham-operated HanSD rats, sham-operated TGR, and ACF TGR, as well as the images from the same groups in the transversely cut hearts. These images show that sham-operated TGR exhibited signs of concentric LV hypertrophy compared to sham-operated HanSD rats (Figures Supplemental 1B and 1E versus Supplemental Fig. 1A and 1D). ACF creation in this phase already tended to produce some dilatation of both LV and RV cavities, with further increases in the wall mass and, notably, left atrial enlargement is clearly visible in the representative images (Supplemental Fig. 1C and 1F).

#### Cardiac structure and function analyzed by echocardiography

As summarized in Table 1, sham-operated TGR compared with sham-operated HanSD rats displayed increased LV anterior and posterior wall thickness without significantly changing LV diameter, leading to profound increase in relative wall thickness (by 56%), which indicated concentric LV hypertrophy and confirmed the data from gross morphological analysis (Supplemental Fig. 1). Otherwise sham-operated TGR did not show any signs of LV systolic or diastolic dysfunction as compared with sham-operated HanSD rats. Compared with sham-operated TGR, dilatation of the LV in ACF TGR echocardiography indicated eccentric remodeling with relative wall thinning (relative wall thickness was reduced by -52%) and also indicated LV systolic dysfunction (LV ejection fraction and LV fractional shortening were reduced by -10% and -20%, respectively). ACF TGR also displayed marked dilatation of the left atrium (by 109%) as compared with sham-operated TGR. ACF TGR also showed signs of impaired diastolic function as seen from the increased LV velocity of E-wave and the ratio of E-wave to A-wave as well as the ratio of E/E^´^ waves – the features clearly different from those in sham-operated TGR. Moreover, echocardiography also showed that RV was markedly dilated in ACF TGR compared to sham-operated TGR. However, despite a clear tendency to lower RV fractional change in ACF TGR, the difference did not reach statistical significance, suggesting that RV systolic function was not yet significantly impaired in ACF TGR compared to sham-operated TGR. ACF TGR showed a marked increase in the vena cava inferior diameter compared to sham-operated TGR and sham-operated HanSD rats, confirming that substantial blood recirculation occurred via ACF.

**Table 1:** Echocardiographic analysis of cardiac structure and function performed 2 weeks after creation of the aorto-caval fistula or sham-operation

	HanSD	TGR	ACF TGR
Heart rate (min^−1^)	357 ± 17	349 ± 12	332 ± 9
Left ventricle diastolic diameter (mm)	6.64 ± 0.20	6.69 ± 0.19	9.95 ± 0.19^@^
Left ventricle systolic diameter (mm)	3.08 ± 0.24	3.15 ± 0.23	5.37 ± 0.27^@^
Left ventricle anterior wall thickness in diastole (mm)	1.57 ± 0.06	2.52 ± 0.09^*^	1.77 ± 0.07
Left ventricle posterior wall thickness in diastole (mm)	1.78 ± 0.04	2.41 ± 0.11^*^	2.03 ± 0.05
Left ventricle relative wall thickness	0.539 ± 0.03	0.842 ± 0.06^*^	0.406 ± 0.02^@^
Left ventricle ejection fraction (%)	83.7 ± 2.2	83.1 ± 2.2	75.5 ± 2.7^@^
Left ventricle fractional shortening (%)	57.4 ± 2.1	56.3 ± 2.5	45.1 ± 2.1^@^
Left ventricle stroke volume (µl)	187.9 ± 9.3	192.9 ± 13.2	402.9 ± 17.1^@^
Cardiac output (ml.min^−1^)	66.3 ± 2.5	70.8 ± 3.2	164.1 ± 6.3^@^
Left atrium diameter (mm)	3.57 ± 0.14	3.48 ± 0.13	7.27 ± 0.26^@^
Left ventricle E Velocity (m.s^−1^)	0.93 ± 0.05	0.89 ± 0.04	1.42 ± 0.06^@^
Left ventricle E/A Ratio	11.9 ± 0.78	16.6 ± 0.76^*^	19.9 ± 0.43^@^
Left ventricle E/E´ Ratio	1.21 ± 0.03	1.18 ± 0.04	1.61 ± 0.09^@^
Right ventricle basal diameter in diastole (mm)	3.44 ± 0.11	3.36 ± 0.18	5.97 ± 0.26^@^
Right ventricle midcavity diameter in diastole (mm)	3.08 ± 0.15	3.17 ± 0.13	5.39 ± 0.24^@^
Right ventricle diastolic area (mm^2^)	27.74 ± 2.75	27.53 ± 1.43	64.99 ± 4.51^@^
Right ventricle systolic area (mm^2^)	15.44 ± 1.55	15.01 ± 2.04	40.52 ± 2.93^@^
Right ventricle fractional change (%)	44.16 ± 2.81	45.8 ± 2.38	37.41 ± 2.31
Diameter of Vena Cava Inferior (mm)	2.35 ± 0.19	2.31 ± 0.14	5.63 ± 0.34^@^

The values are means ± SEM. TGR, Ren-2 renin transgenic rats; HanSD, Hannover-Sprague transgene-negative rats; ACF, aorto-caval fistula. ^*^p < 0.05 TGR versus HanSD ^**@**^*p* < 0.05 ACF TGR versus all other groups

*E* E-wave represents the velocity of blood flow during the rapid passive filling phase of the left ventricle; *A* A-wave represents the velocity of blood flow during the active filling phase of the left ventricle, which occurs after the contraction of the left atrium; *E*′ the maximum velocity of mitral annular movement during the diastole

#### Cardiac function assessed by invasive hemodynamics and PV analysis

**Fig. 3:**
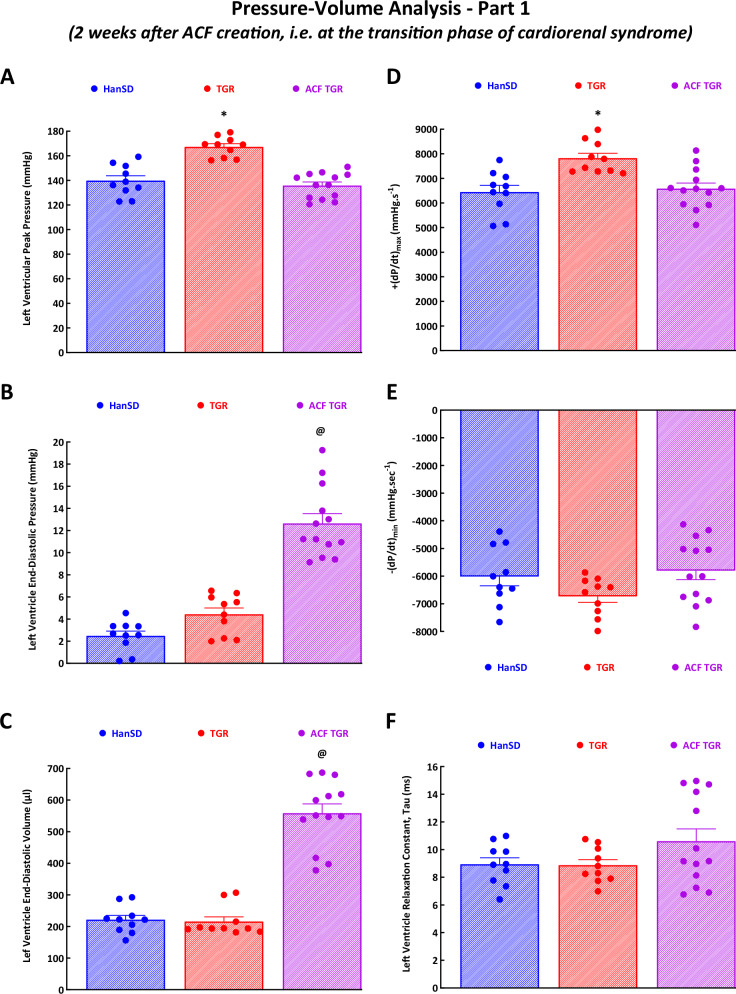
Part 1 of the left ventricular cardiac function assessment by invasive hemodynamic analysis performed in sham-operated normotensive transgene-negative Hannover Sprague-Dawley rats (HanSD), hypertensive sham-operated Ren-2 transgenic (TGR) rats and in TGR two weeks after creation of the aorto-caval fistula (ACF). The data show left ventricle peak pressure **A**, left ventricle end-diastolic pressure **B**, left ventricle end-diastolic volume **C**, maximum rates of pressure rise ( + dP/dt)_max_ **D**, maximum rates of pressure fall (-dP/dt)_min_ **E** and relaxation constant tau of the left ventricle **F**. ^*^ *P* < 0.05 compared with sham-operated HanSD rats. ^**@**^ *P* < 0.05 compared with all other groups. The values are means ± SEM

**Fig. 4:**
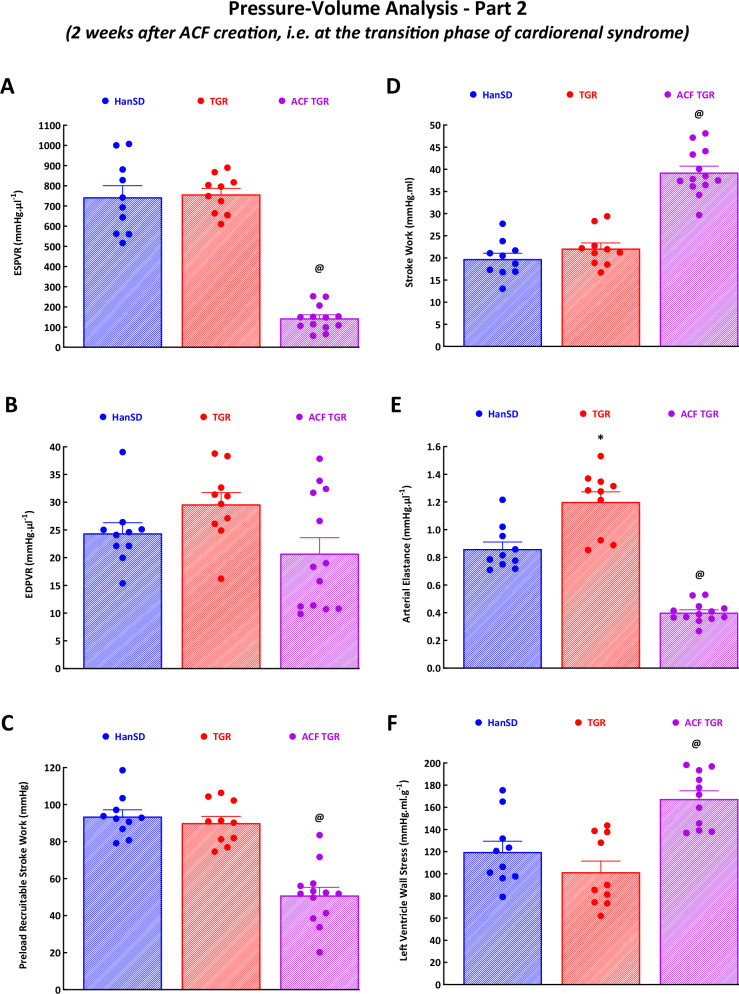
Part 2 of the left ventricular cardiac function assessment by invasive hemodynamic analysis performed in sham-operated normotensive transgene-negative Hannover Sprague-Dawley rats (HanSD), hypertensive sham-operated Ren-2 transgenic (TGR) rats and in TGR two weeks after creation of the aorto-caval fistula (ACF). Shown are: end-systolic pressure-volume relationship (ESPVR) **A**, end-diastolic pressure-volume relationship (EDPVR) **B**, preload recruitable stroke work **C**, stroke work **D**, arterial elastance **E** and left ventricle wall stress **F**. ^*^ *P* < 0.05 compared with sham-operated HanSD rats. ^**@**^ *P* < 0.05 compared with all other groups. The values are means ± SEM

Figures 3 and 4 and Supplemental Fig. 2 present the evaluation of cardiac function by the invasive hemodynamics method. Sham-operated TGR displayed higher LV peak pressure (Fig. 3A), maximum rates of pressure rise ( + dP/dt)_max_ (Fig. 3D), and arterial elastance (Fig. 4E) as compared with sham-operated HanSD rats; however, for all other parameters, there were no significant differences indicating that sham-operated TGR showed signs typical for hypertensive individuals in the compensation phase of hypertension. This notion is further supported by the finding that sham-operated TGR displayed similar VAC as that observed in sham-operated HanSD rats (Supplemental Fig. 2A). However, ventricular efficiency was already slightly impaired in sham-operated TGR as compared with sham-operated HanSD rats (Supplemental Fig. 2D).

ACF TGR displayed significantly lower LV peak pressure (Fig. 3A), ( + dP/dt)_max_ (Fig. 3D), ESPVR (Fig. 4A), PRSW (Fig. 4C) and arterial elastance (Fig. 4E) as compared with sham-operated TGR. In addition, ACF TGR showed higher LV end-diastolic pressure (Fig. 3B), LV end-diastolic volume (Fig. 3C), stroke work (Fig. 4D), and LV wall stress (Fig. 4F) as compared with sham-operated TGR. Moreover, ACF TGR displayed markedly elevated VAC, PE and PVA as compared with sham-operated TGR (Supplemental Fig. 2A–2C), resulting in profound decreases in ventricular efficiency (Supplemental Fig. 2D) in ACF TGR. There were no significant differences in (-dP/dt)_min_ (Fig. 3E), LV relaxation constant, Tau (Fig. 3F) and EDPVR (Fig. 4B) between experimental groups.

### Histological evaluation of heart tissue at the transition phase of cardiorenal syndrome

#### Morphological changes at the cardiomyocyte level (Fig. 5A–C)

**Fig. 5:**
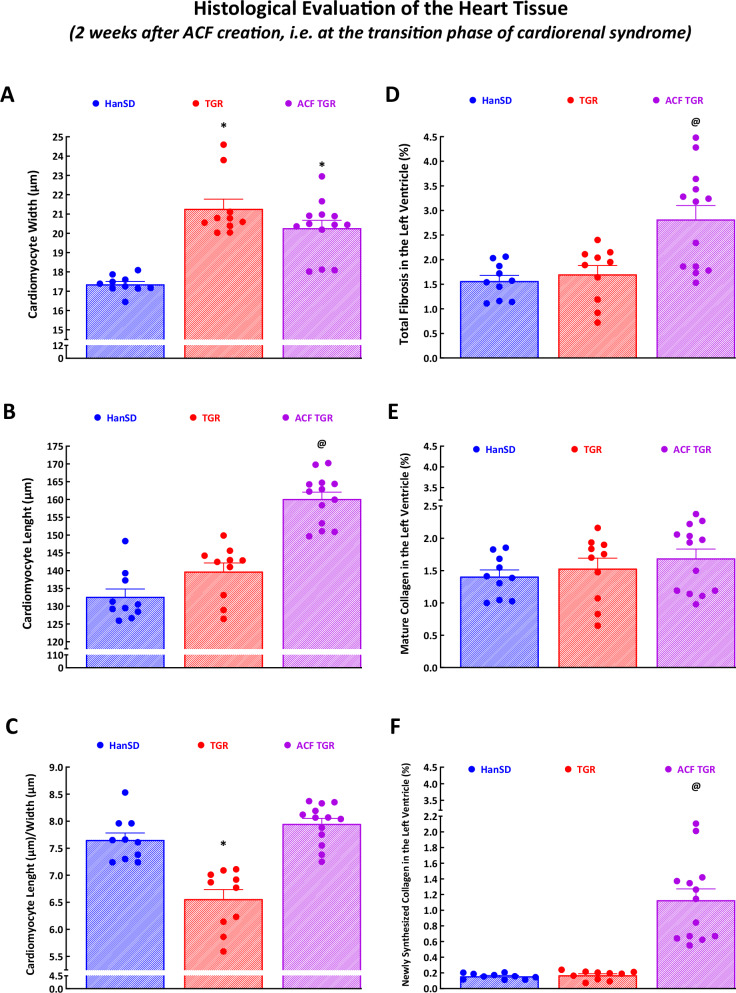
Histological evaluation. Cardiomyocyte width **A**, cardiomyocyte length **B**, the ratio of cardiomyocyte length to cardiomyocyte width **C**, the total fibrosis in the left ventricle **D**, the amount of mature collagen in the left ventricle **E** and the amount of newly synthesized collagen in the left ventricle **F** - in sham-operated normotensive transgene-negative Hannover Sprague-Dawley rats (HanSD), hypertensive sham-operated Ren-2 transgenic (TGR) rats and in TGR two weeks after creation of the aorto-caval fistula (ACF). ^*^ *P* < 0.05 compared with sham-operated HanSD rats. ^**@**^ *P* < 0.05 compared with all other groups. The values are means ± SEM

Figure 5 A–C shows that ACF TGR showed similar cardiomyocyte width as observed in sham-operated TGR; these values were significantly higher than in HanSD rats (Fig. 5A), whereas ACF TGR showed cardiomyocyte length higher than sham-operated TGR and sham-operated HanSD rats; this resulted in a higher ratio of cardiomyocyte length to cardiomyocyte width in ACF TGR as compared with sham-operated TGR (Fig. 5C). These findings corroborate the observation from the whole organ level (Fig. 2 and Supplemental Fig. 1) that ACF TGR showed signs of eccentric LV hypertrophy.

#### Myocardial fibrosis (Fig. 5D–F)

**Fig. 6:**
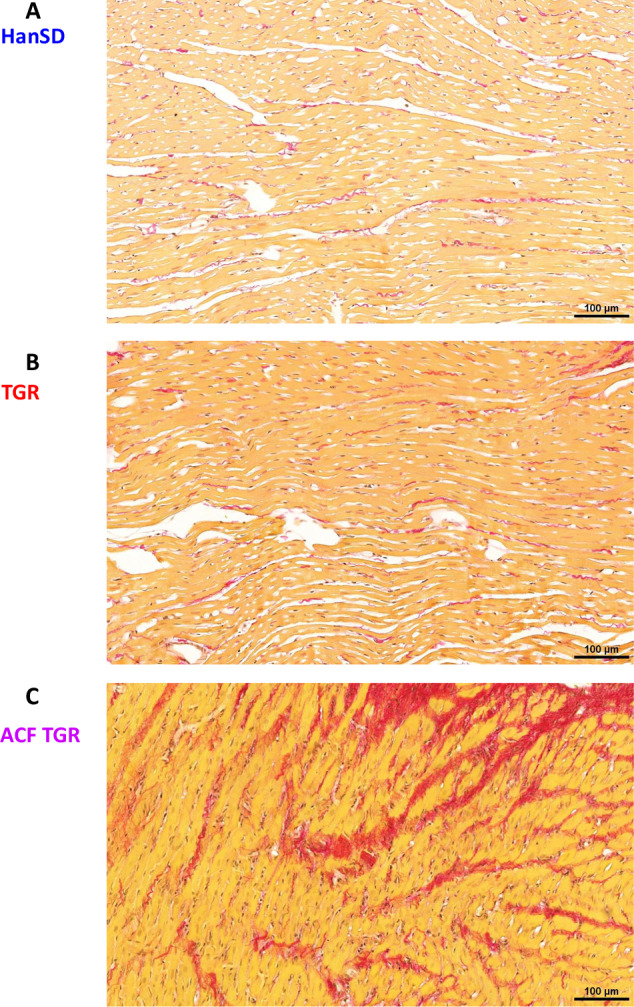
Representative histological images of the left ventricle. Sections are stained with Picrosirius Red (200x), in these bright-field microscopy images, the collagen is red against a pale-yellow background. The scale bar in the figure is 100 µm. **A** sham-operated normotensive transgene-negative Hannover Sprague-Dawley rats (HanSD) (average fibrosis in this group is 1.57 ± 0.11%), **B** hypertensive sham-operated Ren-2 transgenic (TGR) rats (average fibrosis in this group is 1.70 ± 0.18%) and **C** in TGR two weeks after creation of the aorto-caval fistula (ACF) (average fibrosis in this group is 2.83 ± 0.26%)

There were no significant differences in total LV myocardial fibrosis (expressed in %) between sham-operated HanSD rats and sham-operated TGR. However, ACF TGR displayed significantly higher total myocardial fibrosis than observed in both sham-operated groups of animals (Fig. 5D). Notably, there were no significant differences in the content of mature collagen between experimental groups (Fig. 5E), but ACF TGR showed markedly more of newly synthesized collagen as compared with sham-operated groups of animals (Fig. 5F). These data strongly indicate that the increase in the total LV myocardial fibrosis in ACF TGR is mainly mediated by increased collagen formation after ACF creation. Representative images of myocardial fibrosis in the LV are shown in Fig. 6.

### Assessment of LV mRNA expression at the transition phase of cardiorenal syndrome

#### Gene mRNA expression analysis of cardiac markers of stress and contractility

**Fig. 7:**
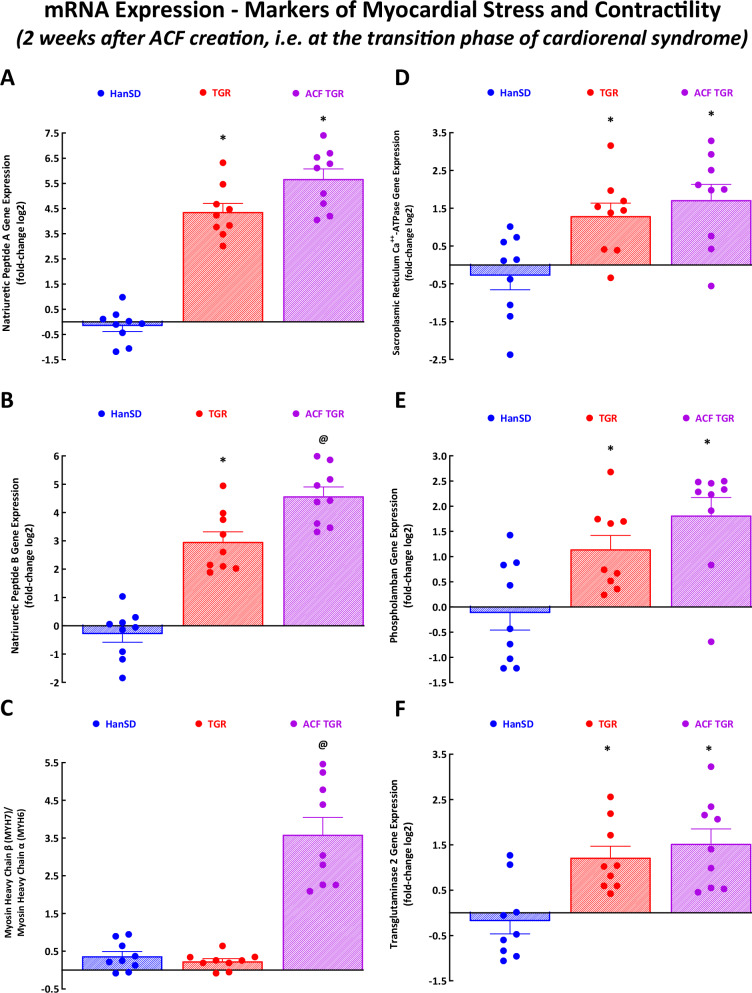
The first part of the data for mRNA expression in the left ventricle – gene markers of myocardial stress and contractile function. Natriuretic peptide A **A**, natriuretic peptide B **B**, the ratio of the β-myosin heavy chain (Myh7) to α-myosin heavy chain (Myh6) **C**, sarcoplasmic reticulum Ca^2+^-ATPase **D**, phospholamban **E** and transglutaminase 2 **F** in sham-operated normotensive transgene-negative Hannover Sprague-Dawley rats (HanSD), hypertensive sham-operated Ren-2 transgenic (TGR) rats, and in TGR assessed two weeks after creation of the aorto-caval fistula (ACF). ^*^ *P* < 0.05 compared with sham-operated HanSD rats. ^**@**^ *P* < 0.05 compared with all other groups. The values are means ± SEM

As shown in Fig. 7, sham-operated TGR and ACF TGR displayed increased mRNA expression of myocardial stress genes as compared with sham-operated HanSD rats: natriuretic peptide type A (Nppa) (Fig. 7A), natriuretic peptide type B (Nppb) (it was more pronounced in ACF TGR than in sham-operated TGR) (Fig. 7B) and transglutaminase-2 (Tgm2) (Fig. 7F). In addition, sham-operated TGR and ACF TGR displayed increased mRNA expression of myocardial genes related to the process of contraction and relaxation: this was so with sarcoplasmic reticulum Ca^++^-ATPase (SERCA) (Fig. 7D) and phospholamban (Pln) (Fig. 7E). ACF TGR showed an increased ratio of mRNA expression of myosin heavy chain beta (MYH7) to myosin heavy chain alfa (MYH6); this resulted from the combined upregulation of MYH7 and downregulation of MYH6 (Fig. 7C).

#### Gene mRNA expression of cardiac markers of metabolism

**Fig. 8:**
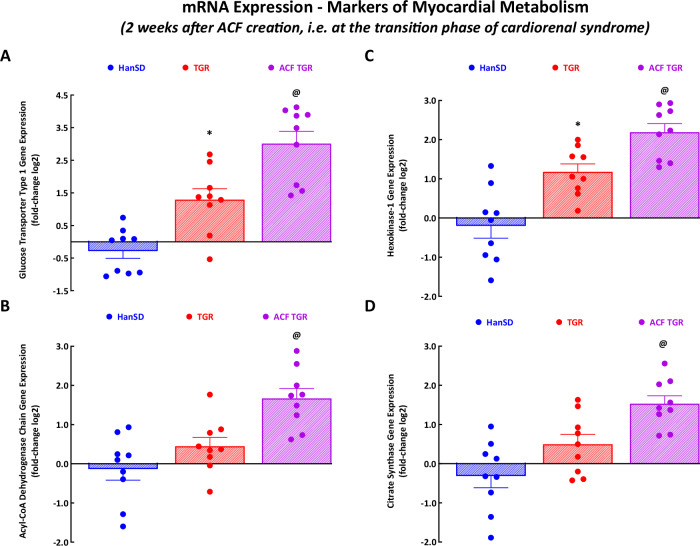
The second part of the data for mRNA expression in the left ventricle – gene markers of myocardial metabolism. Glucose transport type 1 **A**, acyl-CoA dehydrogenase, C-4 to C-12 straight chain **B**, hexokinase-1 **C**, and citrate synthase **D** in sham-operated normotensive transgene-negative Hannover Sprague-Dawley rats (HanSD), hypertensive sham-operated Ren-2 transgenic (TGR) rats, and in TGR two weeks after the creation of the aorto-caval fistula (ACF). ^*^ *P* < 0.05 compared with sham-operated HanSD rats. ^**@**^ *P* < 0.05 compared with all other groups. The values are means ± SEM

As shown in Fig. 8, ACF TGR displayed increased mRNA expression of myocardial substrate metabolism genes as compared with sham-operated HanSD rats as well as sham-operated TGR: this was so in the case of glucose transporter type 1 (GLUT1) (Fig. 8A), Acyl CoA medium chain dehydrogenase (Acadm) (Fig. 8B), hexokinase 1 (Hk1) (Fig. 8C) and citrate synthase (Cs) (Fig. 8D). The mRNA expression of GLUT1 and Hk1 also significantly increased in sham-operated TGR compared with sham-operated HanSD rats (Fig. 8A and 8C).

#### Gene mRNA expression of cardiac markers of inflammation and fibrosis

As shown in Supplemental Fig. 3, sham-operated TGR and ACF TGR displayed increased mRNA expression of myocardial inflammation and fibrosis genes as compared with sham-operated HanSD rats: this was so in the case of interleukin 6 (IL-6), transforming growth factor beta (Tgfb1), collagen type 1, alpha 1 (Col1a1) and collagen type 3, alpha 1 (Col3a1). However, there were no significant differences in mRNA expression of myocardial inflammation and fibrosis gene between sham-operated TGR and ACF TGR.

### Overview of changes in lung and heart weights and cardiomyocyte parameters from the compensation phase (i.e. one week after ACF creation), via transition phase (i.e. two weeks after ACF creation) to the decompensation phase (i.e. three weeks after ACF creation) of cardiorenal syndrome

Below, we present percent changes in organ weights and cardiomyocyte parameters in ACF TGR compared to sham-operated TGR at the same time. The data for the compensation phase and the decompensation phase of cardiorenal syndrome are taken from our previous studies published in Hypertension Research [36, 37] and the data for the transition phase are taken from the current study.

#### Changes in organ weights

Changes are expressed as percent increases in ACF TGR compared to sham-operated TGR at the same time (sham-operated TGR represent 100% basal value).

**Fig. 9:**
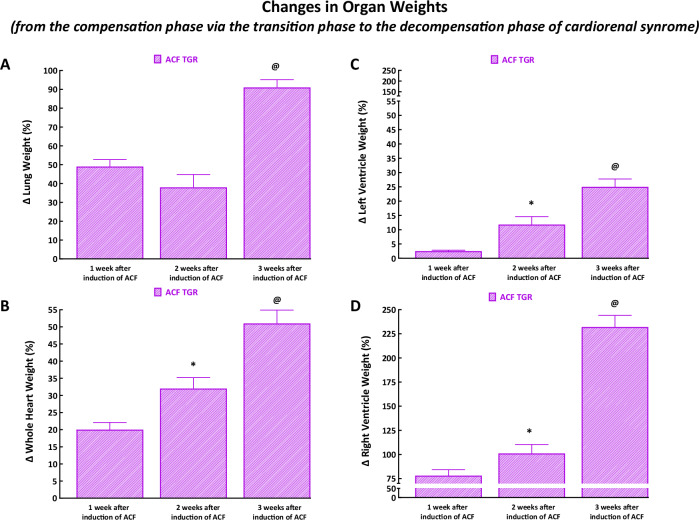
Changes in organ weights from the compensation phase [one week after aorto-caval fistula (ACF) creation, data taken from reference #36], via transition phase (two weeks after ACF creation, data taken from the current study) to the decompensation phase (three weeks after ACF creation, data taken from reference #37). Changes are expressed as percent increases in hypertensive Ren-2 transgenic (TGR) rats with ACF compared to sham-operated TGR at the same time. Changes in lung weight **A**, whole heart weight **B**, left ventricle weight **C**, and right ventricle weight **D**. ^*^ *P* < 0.05 compared with ACF TGR one week after ACF induction. ^**@**^ *P* < 0.05 compared with ACF TGR one week as well as two weeks after ACF induction. The values are means ± SEM

As shown in Fig. 9A, there were similar increases in lung weight one and two weeks after ACF creation, but three weeks after ACF, the TGR displayed an almost twice higher increase in lung weight as compared with the previous phases.

As shown in Fig. 9B, there were progressive increases in whole HW across the three phases of cardiorenal syndrome. However, the simultaneous increases in LVW were relatively modest ( + 2.5 ± 0.3%, +12 ± 3% and +25 ± 3%, respectively) (Fig. 9C), and the increases in whole HW were dominantly mediated (in all three phases) by increases in RVW (by 78 ± 6%, 101 ± 9% and 232 ± 12%, respectively) (Fig. 9D).

#### Changes in cardiomyocyte parameters

**Fig. 10:**
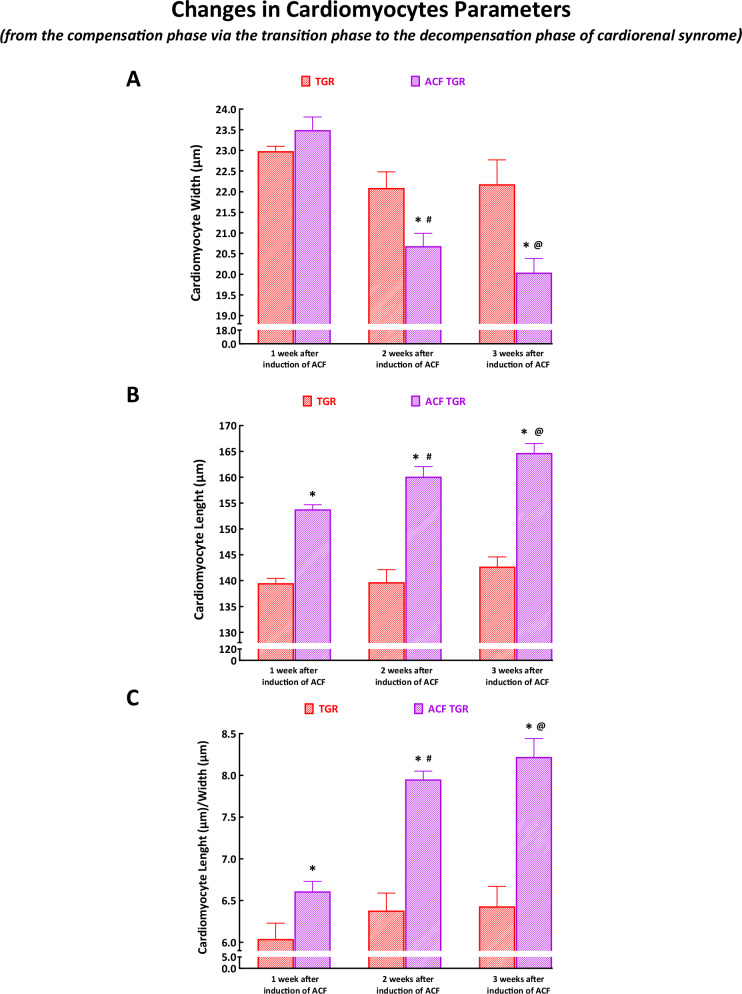
Cardiomyocyte parameters from the compensation phase [one week after aorto-caval fistula (ACF) creation, data taken from reference #36], via transition phase (two weeks after ACF creation, data taken from the current study) to the decompensation phase (three weeks after ACF creation, data from reference #37). Cardiomyocyte width **A**, cardiomyocyte length **B**, and the ratio of cardiomyocyte width to length **C**. ^*^ *P* < 0.05 compared with sham-operated TGR at the same time point. ^**@**^ *P* < 0.05: ACF TGR three weeks after ACF creation versus ACF TGR two weeks after ACF induction. ^**#**^ P < 0.05: ACF TGR two weeks after ACF creation versus ACF TGR one week after ACF creation. The values are means ± SEM

As shown in Fig. 10A, the cardiomyocyte width one week after ACF creation was not significantly different from the values observed in sham-operated TGR. On the other hand, the cardiomyocyte length was already at the beginning significantly higher and progressively increased across all three phases of the cardiorenal syndrome, whereas in sham-operated TGR, it remained stable (Fig. 10B); this resulted in a progressively increasing ratio of cardiomyocyte length to cardiomyocyte width in ACF TGR across all three phases of the cardiorenal syndrome (Fig. 10C).

### Overview of changes in cardiac function assessed by PV analysis from the transition phase (two weeks after ACF creation) to the decompensation phase (three weeks after ACF creation) in cardiorenal syndrome

Below, we present percent changes in cardiac function evaluated by the invasive hemodynamics method in ACF TGR and compare with those seen in sham-operated TGR at the same time. The data for the decompensation phase of cardiorenal syndrome are taken from our previous study published in Hypertension Research [38] and the data for the transition phase are taken from the current study. Percent changes in ACF TGR are shown and compared to sham-operated TGR at the same time (sham-operated TGR again serve as 100% basal value).

**Fig. 11:**
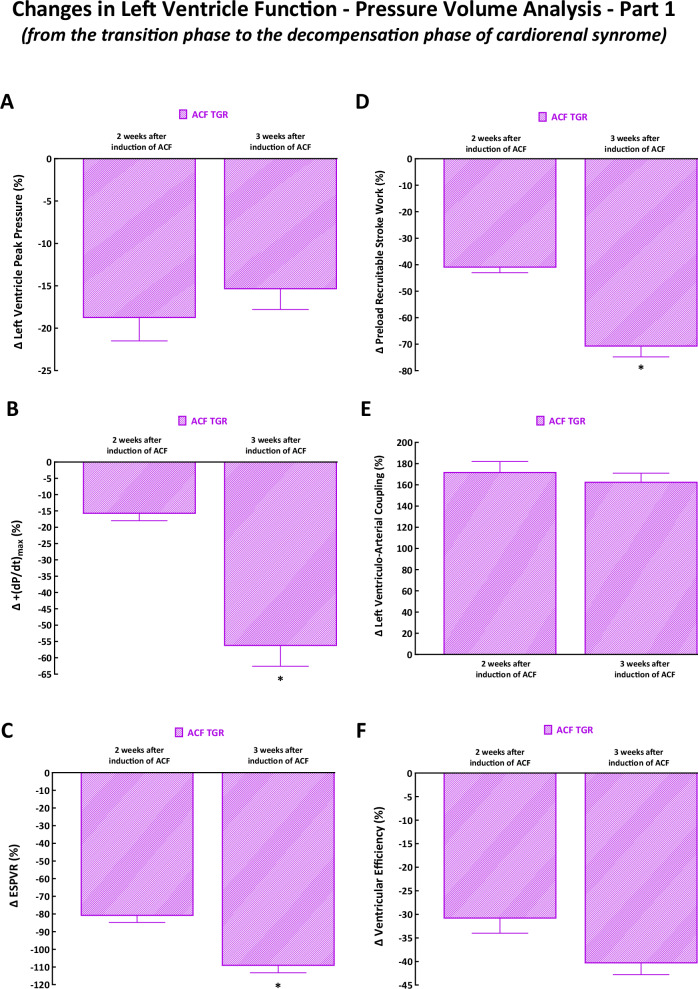
Changes in left cardiac functions (assessed by invasive hemodynamic analysis) from the transition phase (two weeks after ACF creation, data taken from the current study) to the decompensation phase (three weeks after ACF creation, data taken from the reference #37). Changes are expressed as percent increases in hypertensive Ren-2 transgenic (TGR) rats with ACF compared to sham-operated TGR at the same time. Changes are shown in left ventricle peak pressure **A**, maximum rates of pressure rise ( + dP/dt)_max_ **B**, end-systolic pressure volume relationship (ESPVR) **C**, preload recruitable stroke work **D**, left ventriculo-arterial coupling **E** and left ventricular efficiency **F**. ^*^ *P* < 0.05 compared with ACF TGR two weeks after ACF induction

As shown in Fig. 11A, there was a similar decrease in LV peak pressure two and three weeks after ACF creation. Figure 11B–D show that three weeks after ACF creation the TGR displayed significantly higher decreases in ( + dP/dt)_max_, ESPVR and PRSW as compared with the animals two weeks after ACF creation. As shown in Fig. 11E there were similar but especially well-marked increases in VAC two and three weeks after creation. As shown in Fig. 11F, the decreases in LV ventricular efficiency were not statistically different two and three weeks after ACF creation, even though there was a noticeable trend for more pronounced decreases in the latter group.

As summarized in Supplemental Fig. 4, there were no significant differences in the changes in (-dP/dt)_min_, LV relaxation constant, Tau and EDPVR between two and three weeks after ACF creation, anyway these changes were insignificant as compared with the values in sham-operated TGR (Supplemental Fig. 4A–4C). As shown in Supplemental Fig. 4D, the increase in LV wall stress was very pronounced two weeks after ACF creation (confirming the data expressed as absolute values) (Fig. 4F) but LV wall stress significantly decreased three weeks after ACF creation (compared with values observed two weeks after ACF creation, Supplemental Fig. 4D).

## Discussion

The most important findings of our present study can be outlined follows: (i) Two weeks after creation of ACF, ACF TGR showed already fully developed *eccentric* LV hypertrophy, unlike the sham-operated TGR which exhibited signs of LV *concentric* hypertrophy; (ii) The increase in whole HW in ACF TGR was dominantly mediated by RV hypertrophy, whereas the increases in the LV mass were minimal; (iii) Two weeks after ACF creation, the group of ACF TGR did not show any consistent increases in lung weight as compared with sham-operated TGR; (iv) Two weeks after ACF creation, ACF TGR displayed (besides bilateral ventricular dilatation) a significant impairment of LV systolic as well as diastolic functions, whereas RV systolic functions were not impaired; (v) Two weeks after ACF creation, LV of the ACF TGR showed an upregulation of myocardial stress genes, upregulation of the genes reflecting enhanced glycolysis over fatty acid oxidation, and upregulation of the gene responsible for non-insulin dependent glucose uptake in LV.

It is seen that the first important set of findings relates to the organ morphological parameters assessed at the whole organ level as well as at the cardiomyocyte level - in the phase of the transition from the compensation to the decompensation phase of cardiorenal syndrome. Previous studies (including ours) showed that organ weights (whole HW, LVW, RVW, and wet lung weight) are reliable indices of the sudden onset overt HF decompensation, at least in the ACF-induced high-output HF model [33–35, 64–71]. Based on our studies in ACF TGR [36, 37], the rat provides a realistic model of the cardiorenal syndrome we proposed, in accordance with the Linzbach theory [72, 73] (which states that once the myocardial hypertrophic response to the pathological stimuli is exhausted, a progressive ventricular dilatation occurs leading to increased wall stress with subsequent transition of HF to the phase of decompensation), that the inability of LV to develop hypertrophy comparable with the RV might be a critical factor in the transition to the overt decompensation phase of cardiorenal syndrome. For comparative analysis, we employed our present results - obtained exactly in the transition phase of cardiorenal syndrome - and our data obtained earlier in the phase of full compensation and overt decompensation of cardiorenal syndrome [36–38]. As already mentioned in the Introduction, for ethical reasons we did not repeat experiments one week and three weeks after ACF creation and we use the data from our previous studies.

Our results clearly show that increases in LV hypertrophy across all three phases of the cardiorenal syndrome, i.e. from the compensation phase (one week after ACF creation via transition phase - two weeks after ACF creation) to the decompensation phase (three weeks after ACF creation) were modest as compared with increases in RV hypertrophy. Specifically, it is apparent that the RV hypertrophy was already one week after ACF induction 31-fold higher than LV hypertrophy, suggesting that the LV hypertrophic response is substantially delayed in the very early phase of cardiorenal syndrome. Furthermore, the LV hypertrophic response is still considerably hindered in the transition phase and decompensation phase of cardiorenal syndrome: the RV hypertrophy remained nine-fold higher than the LV hypertrophy (Fig. 2C, 2D). However, it is noteworthy that the increase in LVW three weeks after ACF creation was almost twice higher than that observed in ACF TGR two weeks after ACF creation: this likely had a positive effect on the LV wall stress, as discussed below. Moreover, our findings at the cardiomyocyte level clearly showed that the development of eccentric remodeling of the LV dynamically progressed across all three phases of cardiorenal syndrome. Notably, the cardiomyocyte length to cardiomyocyte width ratio increased across all three phases of cardiorenal syndrome; thus, at the transition phase it was already about 25% higher than in sham-operated TGR (Fig. 5C). Furthermore, our present findings regarding the relationship between cardiac hypertrophy and lung congestion are of particular interest. Even though ACF TGR in the transition phase did not show higher lung weight as compared with sham-operated TGR, in three ACF TGR rats, the lung weight was about 70% higher than the average for the group and more than twice higher than observed in sham-operated TGR. Thus, marked lung congestion developed in the said three rats, and this occurred without any significant LV hypertrophy (their LV weight was about 15% lower than the average of the group and was roughly the same as observed in sham-operated TGR) (Fig. 2B and Fig. 2F).

In this context, our findings regarding left atrium diameter (Table 1) and left atrium weight in ACF TGR two weeks after ACF creation are of special interest: they show that the relative absence of LV hypertrophy as compared with sham-operated TGR was accompanied not only by left atrium dilatation, but also by a marked increase in left atrium hypertrophy (particularly obvious in the said three rats that did not develop LV hypertrophy but developed marked lung congestion). Even though the pressure in the left atrium was not measured (enormous technical challenge!) the data suggest that discernable left atrium congestion is in ACF TGR present in the transition phase. Therefore, after a thorough consideration of our previous results and especially of the present morphological data, and taking the Linzbach concept into account [72], we propose that the inability of the LV to develop an appropriate myocardial hypertrophic response to the volume overload in the ACF TGR is a critically important factor underlying the process of the transition from the compensation to the decompensation phase of cardiorenal syndrome. This first interim conclusion is further supported by our second important set of results of echocardiographic and invasive PV analysis of cardiac structure and function.

There were no significant differences in the LV and RV diameters between sham-operated HanSD rats and sham-operated TGR. Nor were there any significant differences between LV and RV function, based on the analysis of LV ejection fraction, the LV fractional shortening, the RV fractional change, the cardiac output, and the parameters of LV filling. All this was corroborated by PV analysis that demonstrated that load-dependent and load-independent parameters of LV contractility [( + dP/dt)_max_, ESPVR, PRSW] were similar in sham-operated HanSD rats and sham-operated TGR, indicating that the latter did not show systolic dysfunction despite significant hypertension and elevated arterial elastance. In addition, there were no significant differences in (-dP/dt)_min_, LV relaxation constant, and the EDPVR between sham-operated HanSD rats and sham-operated TGR, which indicated that sham-operated TGR did not exhibit any important impairment of diastolic function.

These conclusions are confirmed by the findings that the LV end-diastolic pressure and the LV wall stress were not elevated in sham-operated TGR compared with sham-operated HanSD rats. Thus, the concentric LV hypertrophy in sham-operated TGR (increased LV anterior and posterior wall thickness and particularly enhanced relative LV wall thickness) was able to offset the negative consequences of hypertension on the LV function.

In sharp contrast, two weeks after ACF creation, ACF TGR showed markedly increased LV diameter and decreased LV wall thickness (indices of the development of eccentric hypertrophy). Accordingly, there was enhanced LV wall stress, LV end-diastolic pressure, and subsequently impaired LV systolic function (documented by decreased load-dependent and load-independent parameters of the LV contractility). In this context, of particular interest is our observation that ACF TGR in the transition phase did not show significant impairment of the RV systolic function (documented by RV fractional change that was not significantly diminished as compared with sham-operated HanSD rats as well as sham-operated TGR). Admittedly, the values tended to be lower in ACF TGR; however, unlike in the case of the LV, the increase in the RV mass two weeks after ACF creation was prominent. Other interesting findings relate to the diastolic function in ACF TGR observed two weeks after ACF creation: despite increased interstitial collagen content (mainly dependent on the newly synthesized component after ACF creation), and echocardiographic indices of diastolic function (increased E/A and E/E′ ratios in ACF TGR in comparison to TGR and HanSD), ACF TGR did not show significantly impaired (-dP/dt)_min_, LV relaxation constant, and the EDPVR as compared with sham-operated TGR. This suggests that LV stiffness was not markedly enhanced and that ACF TGR in the transition phase may not yet display a pronounced impairment of diastolic function.

Our present data from the transition phase (two weeks after ACF creation) should be considered alongside with our recent data from the decompensation phase (i.e. three weeks after ACF creation) of the cardiorenal syndrome [38]. Comparison of those data shows that ACF TGR in both phases showed insignificant changes in (-dP/dt)_min_, LV relaxation constant, and the EDPVR as compared with sham-operated TGR (Supplemental Fig. 4A–4C). This suggests that impairment of diastolic function is not the critical component in the process of the transition to the decompensation phase of cardiorenal syndrome. In clear contrast is here the comparison of load-dependent and load-independent parameters of LV contractility. Evidently, ( + dp/dt)_max_, ESPVR and PRSW further significantly decreased three weeks after ACF creation as compared with the values seen after two weeks. In fact, and there was a noticeable tendency for further worsening of LV ventricular efficiency (Fig. 11B–D and Fig. 11F). These data suggest that progressing impairment of LV systolic function is importantly involved in the transition to the decompensation phase of cardiorenal syndrome. Therefore, the issue of LV wall stress deserves special attention since it has been long regarded to be an essential determinant of myocardial oxygen consumption and the myocardial contractile status [72].

There is no doubt that the value of LV wall stress reflects in a modified way Laplace´s law and according to the formula presented above in the Methods section it is directly proportional to the pressure and radius and inversely proportional to doubled wall thickness. Therefore, the marked increase in the LV wall stress in ACF TGR observed two weeks after ACF creation (dominantly mediated by the decrease in wall thickness) and the subsequent decrease in the LV wall stress seen three weeks after ACF creation (Supplemental Fig. 4D) is explained by the degree of LV hypertrophy (and LV wall thickness) (Fig. 9C) that was much increased after three weeks.

Our present findings are also in agreement with observations made by Brower and Janicki [68], who, in the model of high-output HF, showed that in ACF HanSD rats, the observed LV hypertrophy exhibits an upper limit: above this, a marked LV dilatation and increased compliance occur. The authors called it “maladaptive ventricular remodeling,” which resulted in overt HF decompensation. Again, such findings are in accordance with the original Linzbach theory [72], which was further developed in detail by the Gerdes group [74, 75]. The above evidence further supports our current notion that the failure of the LV to develop an appropriate hypertrophic response to the volume overload in ACF TGR is likely a crucial factor underlying the process of the transition from the compensation to the decompensation phase of cardiorenal syndrome, and is predominantly due to progressive impairment of LV contractility.

The third important set of findings relates to the analysis of the LV gene expression of selected genes, those implicated in the development of HF [41, 42]. Our results show that ACF TGR displayed upregulation of myocardial stress genes, specifically of NppB and of the ratio of Myh7/Myh6, which suggests that it is linked to LV hemodynamic abnormalities induced by volume overload. In addition, ACF TGR consistently showed increased GLUT1, Acadm, Hk1 and Cs mRNA expression in the LV, which suggested that volume overload induced enhanced insulin-independent glucose uptake and increased glycolysis (compared with fatty acid β-oxidation). The findings are compatible with the activation of the “fetal cardiac gene program” believed to be, in the long-term perspective, maladaptive. Admittedly, the issue remains controversial [76, 77]. However, our correlation analysis clearly showed no difference in the relationship between the critically important markers of the LV cardiac remodeling (LV mass and LV walls stress) and LV mRNA expression of the selected genes of myocardial stress and myocardial metabolism between ACF TGR and sham-operated TGR as well as sham-operated HanSD rats (Supplemental Figs. 5 to 7). This strongly indicates that alterations in gene expression of the selected genes (implicated in the development of HF [41, 42]) are not a causal factor in the transition from the compensation to the decompensation phase of cardiorenal syndrome. The observed gene upregulations in the LV would instead represent a consequence of the onset of cardiorenal syndrome decompensation. It is unknown whether these changes are, in the long-term perspective, adaptive or maladaptive – an issue that could be considered for future studies.

### Limitations of the study

The first important limitation is that the study undertakes evaluation of cardiac structure and function only, even though we employ the term of cardiorenal syndrome, no assessment of renal function was done in this study. As already mentioned in the Introduction the major reason for this omission is that to perform simultaneous evaluation of cardiac function by PV analysis and of renal clearance and hemodynamic studies would be an extraordinary challenge. While our laboratories regularly employ these techniques in separate studies, we cannot so far perform simultaneous evaluation of cardiac and renal function in a way to achieve physiologically reliable results. There is no doubt that such combined studies would require long-term anesthesia and extensive interventional surgical procedures. Nevertheless, we are fully aware that such studies are of critical importance for thorough evaluation of the pathophysiology of cardiorenal syndrome; we believe that in near future we will be able to overcome the technical challenges involved and accomplish simultaneous evaluation of cardiac and renal function in our models of cardiorenal syndrome.

The second limitation of the present study is that it was conducted only in male and since there is increasing recognition that sex should be incorporated as a biological variable in preclinical research being prerequisite for greater reproducibility of research and for successful translation of results into a clinical research [78]. This is particularly valid if TGR are used as a model, because it is known that these rats exhibit important sex-linked differences in the course of hypertension [79, 80] and especially in view of our previous findings that ACF TGR showed important sex-related differences in the course of HF [81, 82]. We are aware of this important limitation and ongoing studies in our laboratories are employing male as well as female animals.

The third limitation of our present study is related to the selection of genes for the analysis of LV mRNA expression as markers of myocardial stress, metabolism, inflammation and fibrosis. These genes were selected based on previous studies in our laboratories [41, 42] that have shown their greatest correlations with changes during RV and LV remodeling. However, it should be noted that those earlier studies were performed under slightly different conditions, even though the same model of ACF-induced HF was employed (NB. only normotensive animals were used). Therefore, our gene selection is not unbiased and may not reflect the full spectrum of transcriptional adaptations in this phase of cardiorenal syndrome. Evidently, further studies are needed to comprehensively analyze transcriptional adaptation to the development of cardiorenal syndrome in this model.

Nonetheless, while admitting the limitations discussed above, we are still convinced that the present results provide important information on the role of “heart side component” in the pathophysiology of experimental high-output HF, a reasonable model of human cardiorenal syndrome.

## Conclusion

Based on our present comprehensive evaluation of cardiac morphology, structure, and function in ACF TGR – a model of high-output HF-induced cardiorenal syndrome - we propose that the inability of the LV to develop appropriate hypertrophic response (leading to maladaptive ventricular remodeling) is a crucial factor in the process of the transition from the compensation to the decompensation phase of cardiorenal syndrome.

Our present findings are important in the light of recent studies that demonstrated that decompensation in our model of the cardiorenal syndrome could not be simply ascribed to impairment of the autoregulatory capacity of renal hemodynamics and the kidney pressure-natriuresis relationship. In fact, we found previously that the pressure-natriuresis relationship was not impaired: it was even improved and facilitated sodium excretion under conditions of lower renal arterial pressure [36, 37]. Taken together, our present and recent findings [36, 37] strengthen the view that to understand the pathophysiology of cardiorenal syndrome (prerequisite for the development of new therapeutic approaches), integrated parallel evaluation of changes in the “kidney side” and in the “heart side” is needed and should be conducted at appropriate phases of this complex syndrome.

## Supplementary information

Supplemental Table 1

Supplemental Figure 1

Supplemental Figure 2

Supplemental Figure 3

Supplemental Figure 4

Supplemental Figure 5

Supplemental Figure 6

Supplemental Figure 7

Supplemental Figure Legends
