# Supplementary material for: The role of ventricular remodeling in the early decompensation of cardiorenal syndrome: Insight from studies with Ren-2 transgenic hypertensive rats subjected to volume overload induced using aorto-caval fistula
Source: Hypertens Res. 2025 Nov 10;49(3):777–800. doi: 10.1038/s41440-025-02440-4 (PMC12960253; doi:10.1038/s41440-025-02440-4)
Supplement: Supplementary file 1 — Supplemental Table 1 [file 41440_2025_2440_MOESM1_ESM.docx]

**Supplemental Table 1.** The genes analyzed in the left ventricle tissue.

**ID Assay Gene Name Abbreviation**

Rn00664637_g1 natriuretic peptide A Nppa

Rn00580641_m1 natriuretic peptide B Nppb

Rn01488777_g1 myosin, heavy chain 7, cardiac muscle, beta Myh7

Rn01488781_g1 myosin, heavy chain 6, cardiac muscle, alpha Myh6

Rn00568762_m1 ATPase, Ca++ transporting, cardiac muscle, slow twitch 2 SERCA

Rn01434045_m1 phospholamban Pln

Rn00571440_m1 transglutaminase-2 Tgm2

Rn01417099_m1 solute carrier family 2 (facilitated glucose transporter), member 1 GLUT1

Rn00566390_m1 acyl-CoA dehydrogenase, C-4 to C-12 straight chain Acadm

Rn00562436_m1 hexokinase 1 Hk1

Rn01774376_g1 citrate synthase Cs

Rn00572711_m1 Interleukin-6 IL-6

Rn00572010_m1 transforming growth factor, beta 1 Tgfb1

Rn01463848_m1 collagen, type I, alpha 1 Col1a1

Rn01437681_m1 collagen, type III, alpha 1 Col3a1

Hs99999901_s1 18S rRNA ribosomal subunit 18s rRNA
