# Supplementary material for: The role of ventricular remodeling in the early decompensation of cardiorenal syndrome: Insight from studies with Ren-2 transgenic hypertensive rats subjected to volume overload induced using aorto-caval fistula
Source: Hypertens Res. 2025 Nov 10;49(3):777–800. doi: 10.1038/s41440-025-02440-4 (PMC12960253; doi:10.1038/s41440-025-02440-4)

Relationship of Left Ventricle Mass and Wall Stress to mRNA Expression of Markers of Myocardial Stress and Contractility - Part 2

(2 weeks after ACF creation, i.e. at the transition phase of cardiorenal syndrome)

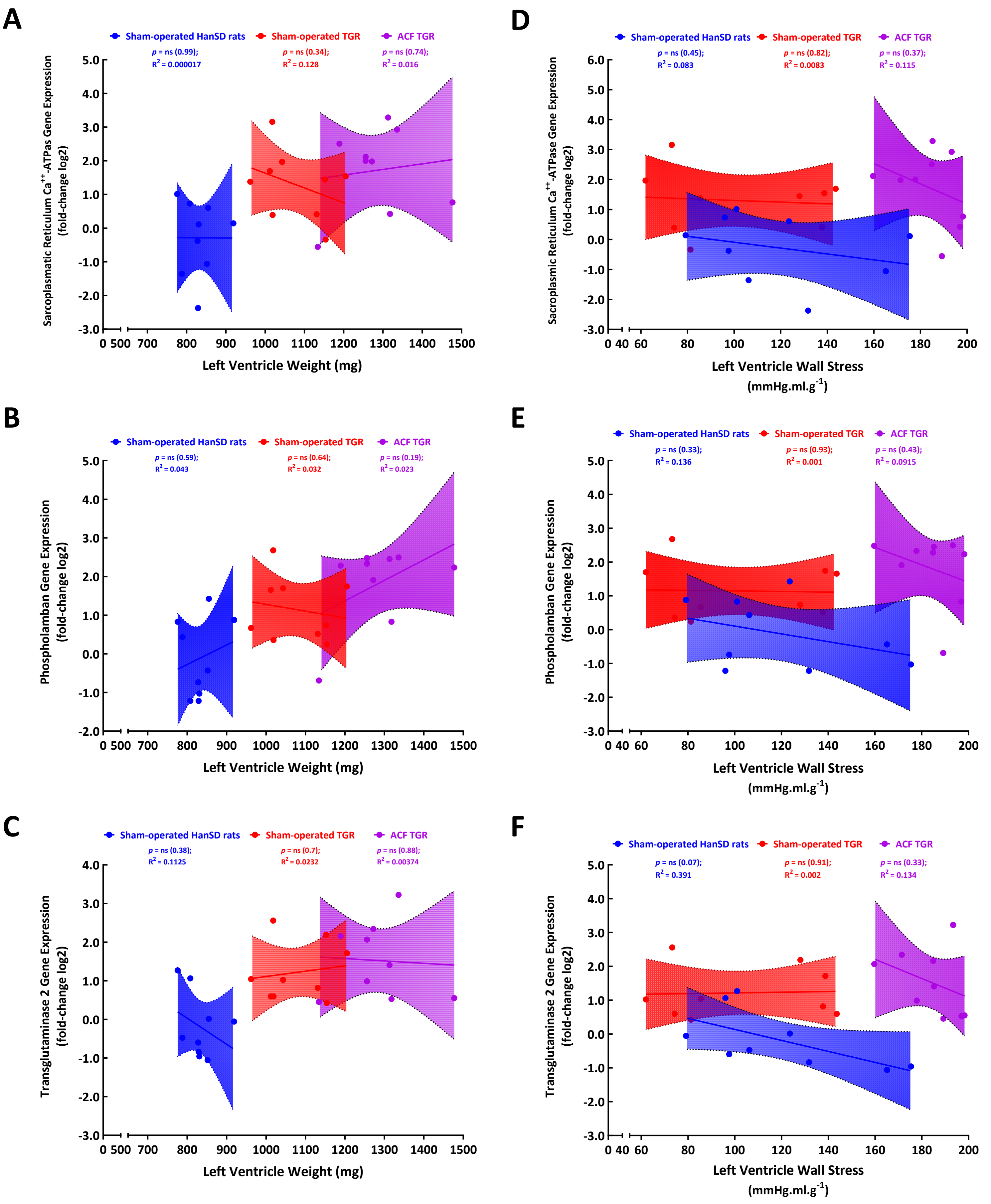

Supplement: Supplementary file 7 — Supplemental Figure 6 [file 41440_2025_2440_MOESM7_ESM.pdf]
