# Supplementary material for: The role of ventricular remodeling in the early decompensation of cardiorenal syndrome: Insight from studies with Ren-2 transgenic hypertensive rats subjected to volume overload induced using aorto-caval fistula
Source: Hypertens Res. 2025 Nov 10;49(3):777–800. doi: 10.1038/s41440-025-02440-4 (PMC12960253; doi:10.1038/s41440-025-02440-4)
Supplement: Supplementary file 9 — Supplemental Figure Legends [file 41440_2025_2440_MOESM9_ESM.docx]

**Supplemental Figure 1.** Representative images of the whole heart and transversally cut heart in (i) sham-operated normotensive transgene-negative Hannover Sprague-Dawley rats (HanSD) (A and D) (average whole heart weight in these groups was 1154 ± 21 mg) and (ii) hypertensive sham-operated Ren-2 transgenic (TGR) rats (B and E); (average whole heart weight in these groups was 1472 ± 39 mg) and in (iii) TGR two weeks after creation of aorto-caval fistula (ACF) (C and F) (average whole heart weight in these groups was 1975 ± 42 mg). The ruler scale is in centimeters (cm) and millimeters (mm).

**Supplemental Figure 2.** Part 3 of the left ventricular cardiac function assessment by invasive hemodynamic analysis performed in sham-operated normotensive transgene-negative Hannover Sprague-Dawley (HanSD), hypertensive sham-operated Ren-2 transgenic (TGR) rats, and in TGR two weeks after creation of the aorto-caval fistula (ACF). The data show ventriculo-arterial coupling (A), potential energy (B), the total pressure-volume area (C) and ventricular efficiency (D). **^*^** P<0.05 compared with sham-operated HanSD rats. **^@^** P<0.05 compared with all other groups. The values are means ± SEM.

**Supplemental Figure 3.** The third part of the data for mRNA expression in the left ventricle – gene markers of inflammation and fibrosis. Interleukin 6 (A), transforming growth factor β (B), collagen type 1, α1 (C) and collagen type 3, α1 (D) in sham-operated normotensive transgene-negative Hannover Sprague-Dawley rats (HanSD), hypertensive sham-operated Ren-2 transgenic (TGR) rats, and in TGR two weeks after creation of the aorto-caval fistula (ACF). **^*^** P<0.05 compared with sham-operated HanSD rats. The values are means ± SEM.

**Supplemental Figure 4.** Changes in left cardiac functions (assessed by invasive hemodynamic analysis) from the transition phase (two weeks after ACF creation, data taken from the current study) to the decompensation phase (three weeks after ACF creation, data taken from the reference #37) Changes are expressed as percent increases in hypertensive Ren-2 transgenic (TGR) rats with ACF compared to sham-operated TGR at the same time. Changes are shown for maximum rates of pressure fall (-dP/dt)_min_ (A), left ventricle relaxation constant, tau (B), end-diastolic pressure-volume relationship (EDPVR) (C) and left ventricle wall stress (D). **^*^** P<0.05 compared with ACF TGR two weeks after ACF induction.

**Supplemental Figure 5.** The first part of the data on the relationship between left ventricle mass and left ventricle wall stress and gene markers of myocardial stress and contractile function. Natriuretic peptide A (A and D), natriuretic peptide B (B and E), and the ratio of the β-myosin heavy chain (Myh7) to α-myosin heavy chain (Myh6) (C and F) in sham-operated normotensive transgene-negative Hannover Sprague-Dawley rats (HanSD), hypertensive sham-operated Ren-2 transgenic (TGR) rats and in TGR two weeks after creation of the aorto-caval fistula (ACF). r: Pearson´s correlation coefficient, p: p-value for correlation.

**Supplemental Figure 6.** The second part of the data on the relationship between left ventricle mass and left ventricle wall stress and gene markers of myocardial stress and contractile function. Sarcoplasmic reticulum Ca^2+^-ATPase (A and D), phospholamban (B and E) and transglutaminase 2 (C and F) in sham-operated normotensive transgene-negative Hannover Sprague-Dawley (HanSD) and hypertensive sham-operated Ren-2 transgenic (TGR) rats and in TGR two weeks after creation of the aorto-caval fistula (ACF). r: Pearson´s correlation coefficient, p: p-value for correlation.

**Supplemental Figure 7.** The relationship of the left ventricle mass and left ventricle wall stress to gene markers of myocardial metabolism. Glucose transport type 1 (A and E), acyl-CoA dehydrogenase, C-4 to C-12 straight chain (B and F), hexokinase-1 (C and G), and citrate synthase (D and H) in sham-operated normotensive transgene-negative Hannover Sprague-Dawley rats (HanSD), hypertensive sham-operated Ren-2 transgenic (TGR) rats, and in TGR two weeks after creation of the aorto-caval fistula (ACF). r: Pearson´s correlation coefficient, p: p-value for correlation.
